# 

**Published:** 1847-07-01

**Authors:** 


### MEDICO-CHIRURGICAL

### REVIEW,

AND

### JOURNAL OF PRACTICAL MEDICINE.

v. 47

NEW SERIES.

VOL. VI.

Not to be indexed

APRIL 1, TO SEPTEMBER 30, 1847.

57487

LONDON:

SAMUEL HIGHLEY, 32, FLEET STREET,

1847.

WI
ME 75T

PRINTED BY F. HAYDEN,
Little College Street, Westminster.

### BIBLIOGRAPHICAL RECORD.

1. The American Journal of Insanity. Edited by the Officers of the New York State Lunatic Asylum, Utica. Parts I. and II. Vol. III. 8vo, pp. 192. Utica, 1846.

2. Statistics of the Royal Infirmary of Glasgow. Third Series. For 1846. By *R. S. Orr,* M.D. 8vo, pp. 36.

3. Thoughts on the Nature and Treatment of several severe Diseases of the Human Body. By *Edward J. Seymour,* M.D. Two Vols. Vol. I. 8vo, pp. 264. London, 1847.

4. The Transactions of the Provincial, Medical, and Surgical Association. Instituted 1832. Vol. III. New Series. 8vo, pp. 437. London, 1847.

5. Remarks on the Diet of Children, and on the Distinctions between the Digestive Powers of the Infant and the Adult. By *G. T. Gream.* 8vo, pp. 201. London, 1847.

6. The Cyclopædia of Anatomy and Physiology. Edited by *Robert B. Todd,* M.D. Part 28. London, 1847.

7. Erect Vision from an Inverted Image. By *B. F. Joslin,* M.D. From the New York Journal of Medicine for November.

8. A Treatise on Fractures in the Vicinity of Joints, and on certain Forms of Accidental and Congenital Dislocations. By *R. N. Smith,* M.D. 8vo, pp. 325. Dublin, 1847.

9. Inhalation of Ether. By *J. Mason Warren,* M.D. 8vo, pp. 18. London, 1847.

10. The Surgeon's Vade Mecum. By *Robert Druitt.* Fourth Edition, 156 Wood-engravings. 8vo, pp. 640. London, 1847.

11. The Construction and Government of Lunatic Asylums and Hospitals for the Insane. By *John Conolly,* M.D. With Plans. 8vo, pp. 191. London, 1847.

12. A Case of Large Secondary Prostatic Calculus removed by Perinæal Incision. From the "Transactions" of the Association. Vol. III. New Series. By *T. Herbert Barker,* M.B. 8vo, pp. 12. London, 1847.

13. Observations on the Connexion between Fever and Famine in Ireland and elsewhere. By *Henry Kennedy,* A.B., &c. 8vo, pp. 50. Dublin, 1847.

*A very able pamphlet, well deserving of attentive perusal.*

14. A Treatise on the Human Ear, with New Views on the Physiology of the Tympanum. By *J. W. Moses*, M.D. 8vo, pp. 18. St. Asaph, 1847.

*Creditable to the ingenuity of the author. His parallel between the cavity of the thorax and the tympanum is cleverly worked out.*

15. Practical Observations on the Pathology and Treatment of certain Diseases of the Skin generally pronounced intractable. By *Thomas Hunt*, M.R.C.S., &c. 8vo, pp. 168. London, 1847.

16. Remarks on Medical Organization and Reform (Foreign and English). By *Edwin Lee.* 8vo, pp. 163. London, 1847.

17. A Treatise on the Structure, Diseases, and Injuries of the Blood-vessels, with Statistical Deductions. Being the Jacksonian Prize Essay for the Year 1844. By *Edwards Crisp*, M.R.C.S., &c. 8vo, pp. 370, with Plates. London, 1847.

18. The Physiological Anatomy and Physiology of Man. By *R. B. Todd,* M.D., and *W. Bowman*, F.R.S. Part III, 8vo. London, 1847.

*In our next.*

19. A System of Surgery. By *J. M. Chelius.* Translated from the German, and accompanied with additional Notes and Observations. By *J. F. South.* Part XVI. 8vo. London, 1847.

20. A Treatise on Diet and Regimen. By *William Henry Robertson*, M.D. Part II. 4th Edition. Re-written and enlarged. 8vo. London, 1847.

21. Observations on Aneurism and its Treatment by Compression. By *O'Bryen Bellingham*, M.D. Edin. 8vo, pp. 189. London.

22. Hydropathy and Homœopathy impartially appreciated, with an Appendix of Notes illustrative of the Influence of the Mind on the Body. By *Edwin Lee*, Esq. The third Editions combined. 8vo, pp. 143. London, 1847.

23. Report of the Health of London Association on the Sanatory Condition of the Metropolis. 8vo, pp. 68. London, 1847.

24. Report of the National Philanthropic Association, instituted March, 1842. 8vo, pp. 30. London, 1847.

25. Vaccination, considered in relation to the Public Health, with Inquiries and Suggestions thereon. A Letter addressed to the Lord Visc. Morpeth. By *John Marshall*, Surgeon. 8vo, pp. 34. London, 1847.

26. The American Journal of the Medical Sciences. Edited by *Isaac Hays*, M D. No. 26, for April, 1847.

27. On Pulmonary Consumption, and on Bronchial and Laryngeal Disease, with Remarks on the Places of Residence chiefly resorted to by the Consumptive Invalid. By Sir *Charles Scudamore*, M.D. 8vo, pp. 278. London.

*In our next.*

28. On Dyspepsia, with Remarks, submitted in support of the Opinion that the Proximate Cause of this, and of all other Diseases affecting the general System, is Vitiation of the Blood. By *John Burdett Steward*, M.D. 8vo, pp. 116. London.

29. On the Pathology and Treatment of Dysentery, being the Gulstonian Lectures delivered at the College of Physicians in February 1847. By *William Baly*, M.D. From the London Medical Gazette. 8vo, pp. 33. London.

*Contain much interesting and valuable information, more especially upon the Dysentery that is endemic in Millbank Prison.*

30. Researches on the Chemistry of Food. By *Justus Liebig*, M.D. Edited by *William Gregory*, M.D. 8vo, pp. 176. London.

*In our next.*

31. On Sir Charles Bell's Researches in the Nervous System. By *Alexander Shaw*, Surgeon of the Middlesex Hospital. Small 4to, pp. 40. London, 1847.

32. Table of Urinary Deposits, with their Tests for Clinical Examination. By *Ray Charles Golding*, M.D.

*Will be useful to the student.*

33. Practical Remarks on the Inhalation of the Vapour of Sulphuric Ether. By *W. Philpott Brookes*, M.D. 8vo, pp. 68. London, 1847.

34. Gazette Medicale, April to July.
*In exchange.*

35. L'Union Medicale, January to July.
*In exchange.*
*Nos.* 58 and 61 *have not arrived.*

36. Dublin Medical Review for May.
*In exchange.*

37. Edinburgh Medical and Surgical Journal for April.

*In exchange.*

38. British and Foreign Medical Review for April.

*In exchange.*

39. Edinburgh Monthly Journal of Medical Science, for April, May, and June.
*In exchange.*

40. Medical Gazette, April to July.
*In exchange.*

